# Diseases of the Throat and Nose, Including the Pharynx, Larynx, Trachea, Œsophagus, Nose, and Naso-Pharynx

**Published:** 1884-11

**Authors:** Frank O. Stockton


### BOOK REVIEWS.

DISEASES OF THE THROAT AND NOSE, INCLUDING THE PHARYNX, LARYNX, TRACHEA, ŒSOPHAGUS, NOSE, AND NASO-PHARYNX. *By* MORELL MACKENZIE, M.D., (*London*) *etc., etc. Volume II. Diseases of the Œsophagus, Nose, and Naso-Pharynx, with Index of Authors and Formulæ for Topical Remedies. Illustrated. Octavo, pp. vi., 550. Philadelphia:* P. BLAKESTON, SON & CO. *1884.*

The second volume for which the profession has been waiting several years. This volume treats of the Œsophagus, Nose, and Naso-pharynx. If there is any difference in the two volumes, it is in favor of the present one. The first portion of Volume II. relates to the disease of the Œsophagus.

The diseases of this organ have been little studied, and it consequently opens a comparatively new field.

In the opening chapter the author shows that the Œsophagoscope is not a plaything, but an instrument of utility, as he has shown that it can be successfully used in the majority of cases.

The chapters on Acute and Chronic Œsophagitis are very interesting, as they treat of diseases that are often overlooked. The other parts of the volume relate to the Nose and Naso-pharynx; they contain nothing particularly new, as it is with this organ that the specialist in this country has particularly to do.

This treatise should have a place in all physicians' libraries, and is also one of the best works for the student, on this subject.

FRANK O. STOCKTON.

